# The Effect of Pain on the Relationship Between Triage Acuity and Emergency Department Hospitalization Rate and Length of Stay

**DOI:** 10.5811/westjem.33600

**Published:** 2025-07-12

**Authors:** Yan-He Lin, Nai-Wen Ku, Chia-Hsin Ko, Eric H. Chou, Chih-Hung Wang, Tsung-Chien Lu, Chien-Hua Huang, Chu-Lin Tsai

**Affiliations:** * College of Medicine, National Taiwan University, Department of Medicine, Taipei, Taiwan; † University of Toronto, Lawrence S. Bloomberg Faculty of Nursing, Toronto, Canada; ‡ National Taiwan University Hospital, Department of Emergency Medicine, Taipei, Taiwan; § College of Medicine, National Taiwan University, Department of Emergency Medicine, Taipei, Taiwan; || Baylor Scott and White All Saints Medical Center, Department of Emergency Medicine, Fort Worth, Texas

## Abstract

**Objectives:**

Little is known about the effect of pain on the relationship between triage and patient outcomes in United States emergency departments (ED). In this study we aimed to describe pain-associated ED visits and to explore how pain modifies the ability of ED triage to predict patient outcomes (hospitalization and ED length of stay [EDLOS)].

**Methods:**

We obtained data from the National Hospital Ambulatory Medical Care Survey (NHAMCS), 2010–2021. Adult ED visits without missing data on pain score or triage level were included. We assessed pain scores at triage using a numeric rating scale (NRS) of 0–10. We further categorized the NRS scores into no (0), mild (1–3), moderate (4–6), and severe (7–10) pain. The five-level Emergency Severity Index was used for ED triage. The primary outcomes were hospital admission during the ED visit and EDLOS. For the analyses we used descriptive statistics and multivariable regression accounting for NHAMCS’s complex survey design.

**Results:**

Over the 12-year study period, there were 132,308 adult ED visits (representing 773,000,000 ED visits nationwide). Approximately 50% were triaged to level 3, followed by 30% to level 4. Approximately 45% reported severe pain, 21% moderate pain, 9% mild pain, and 25% no pain. Triage level 1 was associated with the highest rate of hospitalization (35%), with a gradual decrease in hospitalization rate from levels 2 to 4. Triage level 2 was associated with the longest mean EDLOS (5.6 hours), with a gradual decrease in EDLOS from levels 3 to 5. When stratified by pain intensity, the pattern of hospitalization altered in the mild and moderate pain groups. In these two pain-intensity groups, triage level 1 was associated with lower-than-expected odds of hospitalization, a 31% reduction suggested by the interaction term (adjusted odds ratio 0.69; 95% confidence interval .51–.92, *P* = .01). By contrast, the pattern of EDLOS persisted across all pain-intensity groups.

**Conclusion:**

Mild and moderate levels of pain intensity appear to negatively impact the ability of triage to predict hospitalization, resulting in overtriage among patients in these two pain-intensity groups. Pain intensity in the ED should be carefully evaluated to avoid overtriage and ensure the appropriate allocation of resources.

## INTRODUCTION

Approximately 70–78% of emergency department (ED) visits involve acute pain.^1^–^3^ Thus, pain assessment plays a crucial role in the ED. The American Pain Society introduced pain as a “fifth vital sign” in the 1990s to improve the quality of patient care.^4^ Both the numeric rating scale (NRS) and the visual analog scale are commonly used to measure pain intensity in ED triage; however, these pain scales rely primarily on patients’ self-report.^5^,^6^ Previous studies have shown that pain measurement may not necessarily enhance pain treatment or improve clinical outcomes.^4^,^7^,^8^

**Figure 1:**
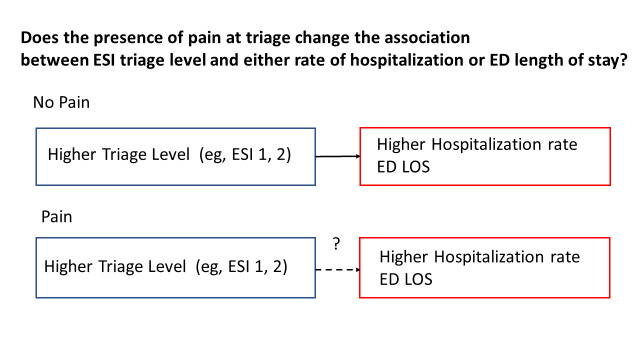
Conceptual diagram of the research question. *ESI*, Emergency Severity Index; *ED*, emergency department; *LOS*, length of stay.

Triage is another crucial component of ED workflow. The ED triage system is used worldwide to identify life-threatening conditions, thereby allocating appropriate resources. The Emergency Severity Index (ESI) in the USA, the Canadian Triage and Acuity Scale (CTAS) in Canada, and the Manchester Triage Scale in the United Kingdom, have been validated against hospital admission, ED length of stay (EDLOS), and resource utilization.^9^–^11^ However, previous research on the CTAS and the Korean Triage and Acuity Scale (KTAS) has raised concerns that using self-reported pain intensity for triage might lead to overtriage.^2^,^12^ Overtriage might lead to prioritizing patients with severe pain over sick patients without a pain complaint. 2,12 To the best of our knowledge, the question of whether pain modulates triage decision-making (ie, pain serving as an effect modifier in the relationship of triage and patient outcomes) in the setting of ESI has not been studied. In epidemiology, effect modification occurs when the effect of a single exposure (ie, triage) on an outcome (eg, hospitalization) depends on the values of another variable (ie, pain). In this context, pain may lead to overtriage (ie, artificially increased triage level without a higher hospitalization rate), modifying the relationship between triage and patient outcomes. The conceptual diagram of our study question is shown in Figure 1.

To fill this knowledge gap, we analyzed 12-year ED data from the National Hospital Ambulatory Medical Care Survey (NHAMCS). Our goal was to describe pain-associated ED visits and explore how pain modifies the ability of the triage system to predict patient outcomes (ie, hospitalization and EDLOS) in the ED.

**Population Health Research Capsule**

What do we already know about this issue?

*Research using triage systems other than the Emergency Severity Index (ESI) has shown that self-reported pain might negatively affect the ability of triage to predict patient outcomes*.

What was the research question?

*We aimed to explore how pain modifies the ability of ED triage to predict hospitalization and ED length of stay*.

What was the major finding of the study?

*A 31% reduction in odds of hospitalization for ESI 1 in mild to moderate pain (adjusted OR, 0.69; 95% CI .51 - .92, P=.01)*.

How does this improve population health?

*The study highlights the importance of meticulous evaluation of pain intensity in the ED, as mild to moderate pain may result in overtriage*.

## METHODS

### Study Design and Setting

The National Hospital Ambulatory Medical Care Survey (NHAMCS) is a cross-sectional, multistage probability sample of visits to non-institutional general and short-stay hospitals located in the 50 US states and the District of Columbia, excluding federal, military, and Veterans Administration hospitals.^13^ The NHAMCS is conducted annually by the National Center for Health Statistics (NCHS). The survey covers geographic primary sampling units, hospitals within primary sampling units, EDs within hospitals, and patients within EDs. The number of EDs sampled is approximately 300–400 per year. Trained research staff collected clinical information during a randomly assigned four-week period for each of the sampled EDs using a structured patient record form (PRF). Data collected included patient demographics, triage level, pain score, reasons for visit, diagnoses, procedures, medications given at the visit, and basic characteristics of the hospital. Quality control was performed using a two-way independent verification procedure for a 10% sample of the records. The non-response rate for most items was less than 5%. The coding error rates were <2%.^14^ Because the NHAMCS contains de-identified data only, our institutional review board exempted this study from review. The study was presented following the Strengthening the Reporting of Observational Studies in Epidemiology (STROBE) guidelines.^15^

### Study Population

We used the NHAMCS data from 2010–2021 in this analysis. First, we excluded ED visits made by patients <18 years of age. We further excluded patient visits with missing data on pain score or triage level. In other words, adult ED visits without missing data on pain score or triage level comprised the study population.

### Variables

Pain scores were assessed at triage using a numeric rating scale (NRS) of 0 to 10. We further categorized the NRS scores into no (0), mild (1–3), moderate (4–6), and severe (7–10) pain. The five-level Emergency Severity Index (ESI) was used for ED triage. To preserve consistency across years, race/ethnicity was recoded as non-Hispanic white, non-Hispanic black, Hispanic, and other. Up to five diagnosis fields in the NHAMCS were coded according to the *International Classification of Diseases*, 9^th^ and 10^th^ Revisions, Clinical Modification. For the current analysis, we used the primary ED diagnosis field when examining triage and pain group-specific diagnoses. Visit disposition was recorded for each ED visit, including admission to the hospital and EDLOS. The EDLOS in the NHAMCS was defined as the time difference between ED triage and ED departure.

### Outcome Measures

The primary outcomes were hospital admission during the ED visit and EDLOS.

### Statistical Analysis

We used Stata 16.0 (StataCorp, College Station, TX) to adjust the variances for the NHAMCS estimates to account for the complex design of the survey. Standard errors (SE) were calculated for the NHAMCS estimates. All statistical tests were based on estimates that had at least 30 cases and a relative SE of <30% (ie, the SE divided by the estimate expressed as a percentage of the estimate) in the sample data, according to the NCHS recommendations. We present descriptive statistics as proportions (with 95% confidence intervals [CI]) or means [with SE]). Multivariable logistic regression analysis was performed to assess the triage level-specific odds ratios (OR) of hospitalization across the pain-intensity groups, adjusting for age, sex, and race/ethnicity. We tested two-way interactions between the pain-intensity groups and triage levels by adding an interaction term in the model. The ORs are presented with 95% CIs. All *P*-values are two-sided, with *P*<.05 considered statistically significant.

## RESULTS

**Figure 2:**
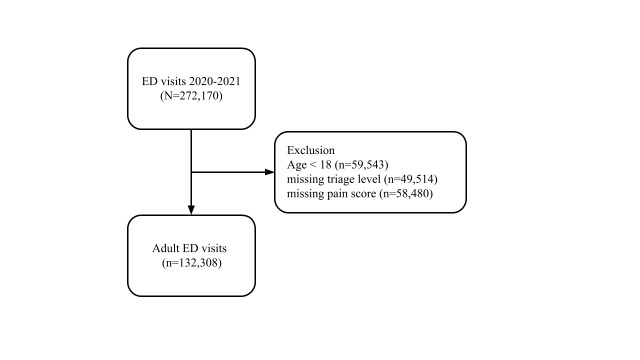
The patient selection process. *ED*, emergency department.

The selection process of the study is presented in Figure 2. From 2010–2021, 272,170 ED visits were recorded in the NHAMCS. After excluding visits made by patients <18 years of age or visits with missing data on triage level or pain score, 132,308 adult ED visits were included in the analysis.

Table 1 demonstrates ED visits by triage level or pain-intensity group. After weighting, a total of 773 million visits were included in this analysis. A total of 7,160,000 (0.9%) patients were triaged to level 1, 98,700,000 (12.8%) to level 2, 396,000,000 (51.2%) to level 3, 235,000,000 (30.4%) to level 4, and 37,000,000 (4.8%) to level 5. Regarding pain score, most patients were in the severe-pain group (350,000,000, 45.2%), followed by the pain-free group (193,000,000, 24.9%), the moderate-pain group (165,000,000, 21.4%), and the mild-pain group (65,600,000, 8.5%). Table 2 shows the proportion of admission and EDLOS stratified by pain-intensity group or triage level. Patients at triage level 1 had the highest proportion of admission (2,485,000, 34.7%), followed by level 2 (31,300,000, 31.7%), level 3 (54,800,000, 13.8%), and level 4 (7,081,000, 3.0%). By contrast, patients triaged at level 2 had the longest average EDLOS of 5.6 hours, followed by level 1 (4.9 hours), level 3 (4.4 hours), level 4 (2.7 hours), and level 5 (2.5 hours). Stratified by the pain-intensity group, the pain-free group had the highest proportion of admission (36,100,000, 18.7%) and the longest mean EDLOS of 4.4 hours. Of note, the severe-pain group had the lowest proportion of hospital admissions.

### Patient outcome #1: Hospital admission

**Figure 3:**
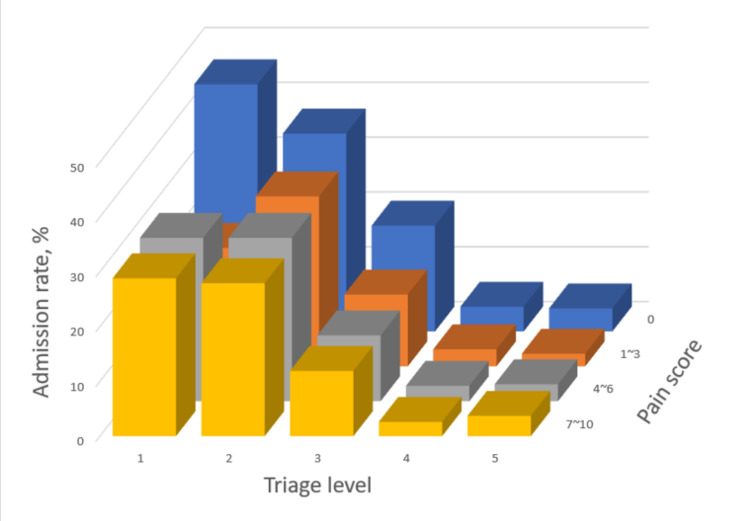
Admission rates associated with triage levels, stratified by the pain-intensity group.

The relationship between triage level and admission rate, stratified by the pain-intensity group is shown in Table 3. In the pain-free group, the triage level 1 patients had the highest admission rate (45.1%), followed by level 2 (36.1%), level 3 (19.3%), and level 4 (4.5%). By contrast, in the mild pain group, triage level 1 patients had a lower admission rate than triage level 2 patients, resulting in a “dip” in the admission rate in Figure 3. Multivariable adjusted odds ratio (aOR) for this subgroup also indicated a less-than-expected likelihood of hospitalization (1.5 for triage level 1 vs 2.4 for triage level 2). In the moderate-pain group, the admission rate for triage level 1 patients was the same as that of level 2 patients, resulting in a “plateau” in the admission rate (not higher than level 2 as one would expect). The interaction term between mild-to-moderate pain and triage level 1 indicated a 31% reduction in the odds of hospitalization (aOR for the interaction term, 0.69; 95% CI .51–.92, *P*=.01). In other words, the effect of triage level 1 on hospitalization was reduced by 31% among patients with mild-to-moderate pain compared with other patients. In the severe-pain group, the patients at triage level 1 still had the highest admission rate of 28.8%, followed by level 2 (27.9%), and level 3 (11.9%).

For the “dip” in admission rate in the mild-pain group, the most common diagnoses for patients who were triaged at level 1 were “open wound of scalp, forehead or hand” (17%) or “chest pain, unspecified” (9%). For the “plateau” of admission rate in the moderate-pain group, the most common diagnoses for patients who were triaged at level 1 were “chest pain, unspecified” (15%) or “head injury, unspecified” (6%).

Pain itself did not increase hospitalization rates (Figure 3), nor was it associated with a higher hospitalization rate at the same triage level. Given the same triage level, the pain-free group consistently had the highest hospitalization rate compared with other pain-intensity groups.

### Patient outcome #2: EDLOS

**Figure 4:**
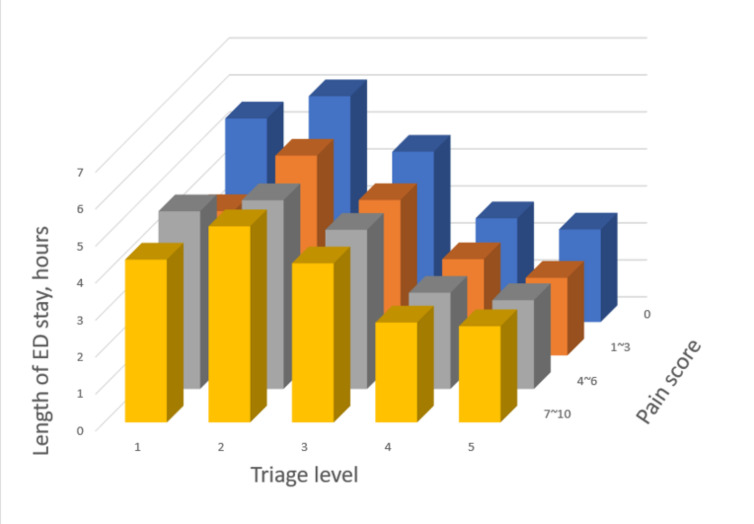
Emergency department length of stay associated with triage levels, stratified by the pain-intensity group. *ED*, Emergency Department.

Table 3 shows how EDLOS changes with triage levels, stratified by the pain-intensity group. All four groups exhibited a consistent pattern, with triage level 2 having the longest EDLOS. For patients triaged at levels 3–5, EDLOS gradually decreased as the triage severity decreased. For example, in the pain-free group, the EDLOS for triage level 2 was 6.1 hours, followed by 4.6 hours at level 3, 2.8 hours at level 4, and 2.5 hours at level 5. The EDLOS for level 1 patients was slightly shorter than that of level 2 patients, except for a deeper drop in EDLOS for level 1 patients in the mild-pain group (Figure 4).

## DISCUSSION

In this study, we analyzed 12-year ED data from the NHAMCS to examine the impact of pain on triage’s ability to predict subsequent patient outcomes. Our analysis showed three major findings: 1) approximately three-quarters of ED visits were associated with pain, representing a major ED subpopulation; 2) pain itself did not increase hospitalization rates; conversely, mild-to-moderate pain intensity appeared to negatively impact the ability of triage to predict hospitalization; and 3) the relationship of triage level with EDLOS was not affected by pain intensity.

### Pain Assessment and Triage Decision

Pain is one of the most common complaints observed in the ED.^16^–^20^ Our results indicated that the national estimate of the prevalence of pain in the ED was approximately 75.1%, a finding that was consistent with previous smaller studies (70–78%).^21^,^22^ Given that pain assessment has become an important part of ED patient evaluation, research has shown that subjective, self-reported pain might interfere with the triage’s predictive validity with respect to patient outcomes. For example, Davis et al analyzed a sample of patients at a tertiary ED and simulated a “pain-free” CTAS for each visit, assuming that the patient had not reported any pain. They found that the removal of the pain scale from CTAS did not reduce its ability to predict hospital admission, intensive care unit (ICU) consultation, or 72-hour mortality.^2^ Moon et al evaluated the triage accuracy using the KTAS by comparing the triage results with those determined by three triage experts. They found a small degree of disagreement between the two, most of which resulted from the misapplication of the pain scale to the KTAS algorithm.^12^ Similarly, Lee et al also reported that the incorporation of pain into KTAS led to an overestimation of patient severity, negatively impacting the predictability of KTAS for urgent patients.^23^ Along these lines, Ku et al also disclosed that self-reported pain seemed to diminish the predictive accuracy of triage for hospitalization in a single-center study. 19 However, those studies were conducted in one or two hospitals without using the ESI for triage purposes. The current study extended the results to US EDs where the ESI is commonly used.

### Hospital Admission Rate

Patients with a higher triage level (eg, level 1) should have a higher admission rate, and this general concept holds true in the pain-free group in our study. However, patients in the mild-pain group exhibited a different pattern, in which the admission rate of triage level 1 patients was lower than expected. This “dip” in admission rate comprised many patients with diagnoses of head/hand wounds and chest pain. Similarly, in the moderate-pain group, many triage level 1 patients had diagnoses of chest pain and head injury. Chest pain can be a dangerous symptom indicative of myocardial infarction. It is likely that in certain circumstances, triage nurses would “err on the side of caution” by triaging patients to level 1, but later these patients were discharged after workup. It may be more difficult to discern the true urgency of chest pain in the mild-to-moderate pain groups than in the severe-pain group, resulting in some degree of overtriage. This held true even when controlling for factors relating to overtriage, such as age, sex, and race/ethnicity,^24^ suggesting that pain itself was a strong factor for overtriage.^25^ Another pain-related factor for overtriage may be traumatic injuries at triage, as open wounds or head injuries were mistriaged to level 1 with relatively low admission rates.^24^

### Emergency Department Length of Stay

Patients triaged to level 2 usually have a longer EDLOS than those triaged to other levels. The possible reason is that patients triaged to triage level 1 have life-threatening conditions and are quickly stabilized and leave the ED for hospitalization. In addition, patients at triage level 2 typically require more examinations and treatments than those at lower triage levels, resulting in a longer EDLOS. Consistent with other ED studies,^26^–^30^ our study showed a similar pattern of EDLOS by triage levels, regardless of pain intensity. This is different from the trend observed in hospitalization. Patients who were mistriaged to level 1 in mild-to-moderate pain groups left the ED sooner due to their lower acuity, preserving the trend of EDLOS by triage levels.

## LIMITATIONS

This study has several limitations. First, various factors can affect pain assessment and triage decisions. Although we attempted to control for patient-level variation in pain expressions, we did not control for nurse-level (eg, experience) and ED-level variations (eg, local triage practice pattern). Despite the extensive data collected by NHAMCS, this nuanced information is still lacking. Notably, the admission rate for ESI 1 patients was 34.7%. This low admission rate may result from overtriage due to pain or local triage practice patterns in smaller EDs. Nonetheless, similar admission rates have been reported in the literature using the same NHAMCS data.^31^ Second, many patients were missing either a triage level or pain score, suggesting that our results should be interpreted with caution due to variations in ED practice. Third, the EDLOS recorded in the NHAMCS was from ED triage to ED departure (as opposed to the decision to admit), and the version of EDLOS was affected by ED boarding. Finally, we excluded patients <18 years of age who may have different ways to express pain. Thus, our results may not be generalizable to children.

## CONCLUSION

In this 12-year study representing 773 million adult ED visits in the US, different levels of pain intensity appear to modulate the ability of triage to predict hospitalization but did not alter the relationship of triage with ED length of stay. The results underscore the importance of meticulous evaluation of pain intensity in the ED setting, as it may negatively impact the predictive validity of a triage system, resulting in overtriage among patients with mild-to-moderate pain. Further research may delve into mechanisms by which pain affects triage ability at various levels, including in patients, nurses, and ED practice patterns. Until the development of an objective tool for pain assessment, training of triage personnel and continuous quality improvement of ED triage may be key. A more standardized and effective triage system would ultimately benefit patients in the ED and improve patient outcomes.

## Figures and Tables

**Table 1:** Emergency department visits stratified by triage level or pain-intensity group.

Variable	Weighted Number	Weighted percentage (95% CI)
Total number of visits	773,000,000	
Triage level, n (%)		
1	7,160,000	0.9 (0.7–1.2)
2	98,700,000	12.8 (12.0–13.5)
3	396,000,000	51.2 (50.1–52.3)
4	235,000,000	30.4 (29.3–31.5)
5	37,000,000	4.8 (4.3–5.3)
Pain-intensity group, n (%)		
No pain (0)	193,000,000	24.9 (24.0–25.8)
Mild (1–3)	65,600,000	8.5 (8.2–8.8)
Moderate (4–6)	165,000,000	21.4 (20.9–21.9)
Severe (7–10)	350,000,000	45.2 (44.3–46.2)

*CI*, confidence interval.

**Table 2:** The proportion of hospital admission and mean emergency department length of stay stratified by triage level or pain-intensity group.

Variable	Weighted proportion of admission N (%)	Weighted EDLOS, mean (95% CI), hour
Triage level, n (%)		
1	2,485,000 (34.7)	4.9 (4.2–5.5)
2	31,300,000 (31.7)	5.6 (5.3–5.8)
3	54,800,000 (13.8)	4.4 (4.2–4.5)
4	7,081,000 (3.0)	2.7 (2.6–2.8)
5	1,325,000 (3.6)	2.5 (2.3–2.7)
Pain score, n (%)		
No pain (0)	36,100,000 (18.7)	4.4 (4.2–4.5)
Mild (1–3)	7,718,000 (11.8)	3.7 (3.5–3.9)
Moderate (4–6)	17,700,000 (10.7)	3.8 (3.7–3.9)
Severe (7–10)	35,400,000 (10.1)	3.8 (3.6–3.9)

*EDLOS*, emergency department length of stay; *CI*, confidence interval.

**Table 3:** Admission rates and emergency department length of stay associated with triage levels stratified by the pain-intensity group.

Variable	Weighted proportion of admission, %	Adjusted odds ratio (95% CI)	Weighted EDLOS, mean (95% CI), hour
No pain (pain score = 0)			
Triage level, n (%)			
1	45.1	3.0 (2.4–3.6)	5.5 (4.4–6.7)
2	36.1	2.3 (2.1–2.4)	6.1 (5.8–6.5)
3	19.3	1.0 (reference)	4.6 (4.5–4.8)
4	4.5	0.3 (0.3–0.3)	2.8 (2.6–3.0)
5	4.2	0.3 (0.2–0.4)	2.5 (2.2–2.7)
Mild pain (pain score = 1–3)			
Triage level, n (%)			
1	21.6	1.5 (0.9–2.4)	3.9 (2.7–5.0)
2	31.0	2.4 (2.1–2.8)	5.4 (4.8–6.0)
3	13.1	1.0 (reference)	4.2 (3.9–4.4)
4	3.1	0.3 (0.2–0.3)	2.6 (2.4–2.8)
5	2.3	0.2 (0.2–0.4)	2.1 (1.8–2.4)
Moderate pain (pain score = 4–6)			
Triage level, n (%)			
1	29.8	2.5 (1.8–3.6)	4.8 (3.4–6.2)
2	29.8	2.5 (2.3–2.8)	5.1 (4.8–5.4)
3	12.0	1.0 (reference)	4.3 (4.2–4.5)
4	2.8	0.3 (0.2–0.3)	2.6 (2.5–2.8)
5	3.1	0.4 (0.3–0.5)	2.4 (1.9–2.8)
Severe pain (pain score = 7–10)			
Triage level, n (%)			
1	28.8	2.7 (2.1–3.4)	4.4 (3.6–5.3)
2	27.9	2.5 (2.3–2.7)	5.3 (5.0–5.6)
3	11.9	1.0 (reference)	4.3 (4.1–4.4)
4	2.6	0.2 (0.2–0.3)	2.7 (2.6–2.8)
5	3.7	0.4 (0.4–0.5)	2.6 (2.3–2.9)

Multivariable model adjusted for age, sex, race/ethnicity, triage level, and pain intensity.

*EDLOS*, emergency department length of stay; *CI*, confidence interval.
